# Liquid Biopsy in Malignant Pleural Mesothelioma: State of the Art, Pitfalls, and Perspectives

**DOI:** 10.3389/fonc.2019.00740

**Published:** 2019-08-14

**Authors:** Ilaria Cavallari, Loredana Urso, Evgeniya Sharova, Giulia Pasello, Vincenzo Ciminale

**Affiliations:** ^1^Immunologia e Diagnostica Molecolare Oncologica (IDMO), Istituto Oncologico Veneto IOV- IRCCS, Padova, Italy; ^2^Department of Surgery, Oncology and Gastroenterology, Università degli Studi di Padova, Padova, Italy

**Keywords:** microRNA, prognostic stratification, early diagnosis, asbestos exposure, liquid biopsy, mesothelioma

## Abstract

Malignant pleural mesothelioma (MPM) is an aggressive tumor linked to asbestos exposure. Although the risk factors for MPM are well-known, the majority of MPM patients are diagnosed at an advanced stage and have a very poor prognosis. Circulating biomarkers for early diagnosis remain to be identified, and the current standard for MPM diagnosis relies on pleural biopsies. Robust non-invasive tests for the screening of asbestos-exposed subjects are therefore an important unmet clinical need. This review provides a critical summary of recent liquid biopsy-based studies aimed at discovering novel blood-based circulating biomarkers for the early diagnosis and prognostic stratification of MPM patients.

## Introduction

Malignant pleural mesothelioma (MPM) is a rare cancer with increasing incidence and dismal prognosis due to its aggressiveness and lack of effective treatments (1–3).

Asbestos exposure is considered the main causative factor for MPM, with a decades-long latency between start of exposure and clinical diagnosis (4). Prolonged exposure to inhaled asbestos fibers trigger an increase in reactive oxygen species (ROS) and inflammatory cytokines in the pleural microenvironment, both of which are key drivers of MPM carcinogenesis (5, 6). However, despite the high ROS burden, MPM is characterized by a low mutation load (7), with tumor suppressors (CDKN2A, BAP1, NF2, LATS2) the most frequently mutated genes involved in MPM pathogenesis (8).

The current standard for the diagnosis and genetic profiling of most tumors involves the use of tissue biopsies (9). However, given its invasive nature, tissue biopsy is burdened with considerable patient morbidity and costs for the health care systems (9, 10). The histopathological diagnosis of pleural biopsies is difficult and may require FISH (fluorescent *in situ* hybridization) of the CDKN2A locus and immunohistochemistry for p16 and BAP1 (11) when invasion is not clearly demonstrated based on the histology, and to confirm the diagnosis of mesothelioma in pleural effusions.

The onset of MPM is insidious and most patients have advanced disease at presentation. Current imaging methods are inadequate for screening and for differential diagnosis of pleural plaques vs. malignant mesothelioma (12). The availability of a robust non-invasive test for the screening of asbestos-exposed subjects is therefore an important unmet clinical need.

**Figure 1:**
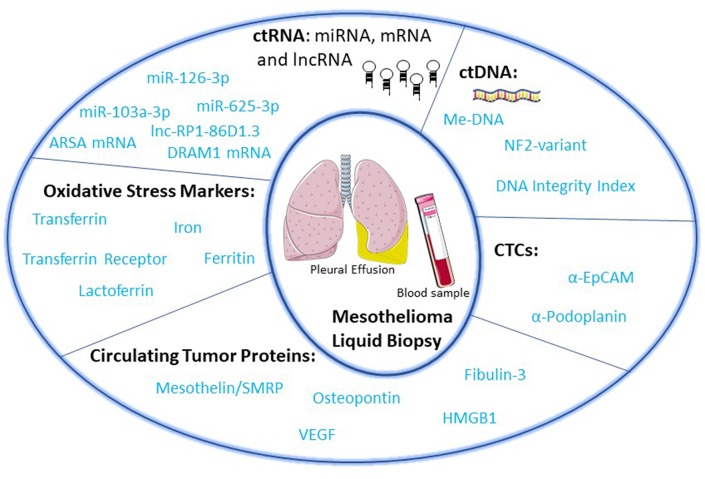
Circulating biomarkers in malignant pleural mesothelioma.

“Liquid biopsy” of biological fluids (e.g., plasma and serum, urine, saliva, cerebrospinal fluid, pleural fluid, ascites, stool) is emerging as a powerful tool for non-invasive diagnosis, screening, prognosis, and stratification of cancer patients. This approach is based on the fact that tumor cells release molecules (proteins, DNA, RNA), circulating tumor cells (CTC), and extra cellular vesicles (EV) that can be used as biomarkers (13) (Figure 1). Liquid biopsies may be repeated frequently to provide a more detailed picture of the natural history of the disease with a longitudinal assessment of tumor burden and clues about clonal evolution and emergence of drug-resistant clones leading to clinical relapse (9, 14).

The following sections provide a critical overview of recent studies describing novel circulating biomarkers for the early diagnosis and prognostic stratification of MPM patients.

## Circulating Tumor Proteins

### Mesothelin

Mesothelin (MSLN) is a cell-surface glycoprotein expressed by mesothelial cells. It is synthesized as a 70-kDa precursor which is cleaved by Furin protease to produce the mature form of Mesothelin and Megakariocyte Potentiating Factor (MPF) (15). Mesothelin is overexpressed in ovarian cancer, pancreatic cancer (15) and MPM, especially in the epithelioid and biphasic subtypes (16).

A soluble form of Mesothelin, named Soluble Mesothelin-related peptide (SMRP), is shed by the tumor cells into the circulation (17–19). Although SMRP is not specific for MPM (17, 20, 21), its role as early biomarker for the screening of asbestos-exposed subjects has been extensively studied (Figure 1, Table 1).

**Table 1:** Circulating protein biomarkers evaluated for early diagnosis in MPM.

**Protein biomarker**	**Study design**	**Method**	**Sample**	**Study results**	**References**
SMRP	AEXP = 40, MPM = 44, ARD = 38, ILD = 92, OC = 38	ELISA	Serum	MPM vs. AEXP cut-off 0.22 nmol/L Sensitivity: 84%, Specificity: 83% MPM vs. PD cut-off 0.22 nmol/L Sensitivity: 84%, Specificity: 100% MPM vs. OC cut-off 0.22 nmol/L Sensitivity: 84%, Specificity: 95%	(22)
	MPM = 60, Mets = 30, ARD = 23	ELISA	Serum	MPM vs. ARD AUC = 0.87 cut-off 0.93 nmol/L Sensitivity: 80% Specificity: 82.6% MPM vs. Mets AUC = 0.693 cut-off 1.85 nmol/L Sensitivity: 58.3% Specificity: 73.3%	(23)
	HC = 54, AEXP = 203, ARD = 130, MPM = 107	ELISA	Serum	MPM vs. All AUC = 0.77 cut-off 1 nmol/L Sensitivity: 68.2% Specificity: 80.5%	(24)
	AEXP = 112, Mets = 43, ARD = 33, MPM = 96	ELISA	Serum	MPM vs. AEXP AUC = 0.866 MPM vs. ARD AUC = 0.719	(25)
	AEXP/ARD = 66, MPM = 90	ELISA	Serum	MPM vs. AEXP/ARD AUC = 0.81 cut-off 1.9 nmol/L Sensitivity: 60% Specificity: 89.2%	(26)
	HC = 48, AEXP = 177, ARD = 101, MPM = 36	ELISA	Serum	MPM vs. All AUC = 0.75 cut-off 0.55 nmol/L Sensitivity: 72% Specificity: 72%	(27)
	HC = 120, AEXP = 123, ARD = 279, MPM = 24	ELISA	Serum	MPM vs. All AUC 0.74 cut-off 1.63 nmol/L Sensitivity: 58% Specificity: 83%	(28)
Osteopontin	AEXP/ARD = 69, FS = 45, MPM = 75	ELISA	Serum	MPM vs. AEXP/ARD AUC = 0.888 cut-off 48.3 ng/ml Sensitivity: 77.6% Specificity: 85.5% Stage I MPM vs. AEXP/ARD AUC = 0.906 cut-off 62.4 ng/ml Sensitivity: 84.6% Specificity: 88.4%	(29)
	AEXP = 112, Mets = 43, ARD = 33, MPM = 96	ELISA	Serum, Plasma	MPM vs. AEXP AUC 0.724 MPM vs. Mets AUC = 0.689 MPM vs. ARD AUC = 0.677	(25)
	AEXP = 93, ARD = 111, MPM = 31	ELISA	Plasma	MPM vs. AEXP/ARD AUC = 0.785	(30)
	ARD = 89, MPM = 66	ELISA	Plasma	MPM vs. ARD AUC = 0.763	(31)
	HC = 120, AEXP = 123, ARD = 279, MPM = 24	ELISA	Serum	MPM vs. All AUC 0.86 cut-off 17.273 nmol/L Sensitivity: 75% Specificity: 86%	(28)
Fibulin-3	AEXP = 136, OC = 93, MPM = 92	ELISA	Plasma	MPM vs. All AUC = 0.99	(32)
	Validation study: AEXP = 96, MPM = 48	ELISA	Plasma	MPM vs. AEXP AUC = 0.87	
	Non-MPM = 56, MPM = 84	ELISA	Plasma	MPM vs. Non-MPM AUC = 0.632	(33)
	ARD = 49, BE = 35, OC = 36, MPM 82	ELISA	Plasma	MPM vs. All AUC = 0.671 Cut-off 52 ng/ml Sensitivity: 22% Specificity: 95%	(34)
Acetylated HMGB1	HC = 20, AEXP = 20, BE = 13, OC = 25, MPM = 22	Mass Spectrometry	Serum	MPM vs. AEXP AUC = 1 Cut-off 2 ng/ml Sensitivity: 100% Specificity: 100% MPM vs. BE/OC AUC = 0.837 Cut-off 9.70 ng/ml Sensitivity: 81.82% Specificity: 89.47%	(35)

*AEXP, healthy asbestos-exposed individuals; MPM, Malignant Mesothelioma Patients; ILD, Inflammatory Lung Disease patients; OC, patients affected by other cancers; Mets, pleural metastasis of carcinomas; HC, non-exposed healthy controls; ARD, benign asbestos-related disease patients; FS, former smoker individuals; BE, patients with benign effusion*.

Almost all of these studies used the U. S. Food and Drug Administration (FDA)-approved Mesomark ELISA assay to detect all soluble forms of the protein. Mesomark is a reliable assay that is not affected by the presence of other molecules (e.g., hemoglobin, triglycerides, bilirubin) (20).

The first study investigating SMRP in the context of MPM showed increased SMRP levels in serum samples from 37 out of 44 MPM patients compared to 40 healthy asbestos-exposed subjects (22). The test also distinguished MPM patients from 18 patients affected by non-neoplastic asbestos-related disease and 122 patients with inflammatory lung diseases or other cancers. Unfortunately, SMRP levels showed low accuracy in identifying patients with sarcomatoid MPM and low tumor size (<1 cm). In this study, the authors did not take into account confounding factors such as age, renal dysfunction, and body mass index (BMI) that may “per se” increase SMRP levels (37–40). Other studies confirmed these results (23, 24, 26–28, 41, 42), but were characterized by high heterogeneity regarding the selection of the control study population and cut-off values (18). A meta-analysis of data from 16 different studies indicated low accuracy for early diagnosis because of low sensitivity. Indeed, low SMRP levels did not exclude the presence of malignancy, especially in early-stage disease (18).

Although SMRP cannot be considered an early diagnostic biomarker for surveillance programs, it seemed to be effective in predicting response to chemotherapy and patient survival. This is an important issue, as quantitative radiological measures are difficult for this cancer. In three prospective studies enrolling, respectively, 96, 107, and 100 MPM patients, high baseline SMRP levels significantly correlated with shorter survival (24, 25, 43). In 2010, Creaney et al. performed a prospective evaluation of serum SMRP levels over time in patients undergoing chemotherapy (*N* = 55). These authors found a strong correlation between radiological responses (measured by CT scans according to modified RECIST criteria) and SMRP variations. Specifically, an increase >25% was associated with progressive disease (PD), a decrease >25% with partial response (PR), and no changes with stable disease (SD). Log-rank analysis showed that a decrease in SMRP was strongly associated with longer survival (44). The results were confirmed in another study, although the authors measured SMRP in the plasma and set the cut-off at 10% variation (45). These studies have some limitations such as the small sample size and the heterogeneity of the treatments received; nevertheless, the usefulness of SMRP as an indicator of tumor response deserves further investigation.

Anti-Mesothelin antibodies (e.g., immunotoxin SS1P) are being tested for MPM and other cancers. In these patients MPF (Megakariocyte Potentiating Factor) may be used as a biomarker to evaluate response to therapy as it does not bind the therapeutic antibodies (46). Serum MPF was analyzed in patients enrolled in two clinical trials evaluating SS1P efficacy: a phase I trial tested first line treatment with SS1P in combination with Cisplatin/Pemetrexed (47) and a pilot study investigated SS1P in association with cyclophosphamide and pentostatin in previously treated patients (48); results showed that all patients who experienced PR showed a strong decrease in MPF expression, suggesting that serum MPF might predict clinical outcome (49). However, these studies were carried out on a low number of patients.

### Osteopontin

Osteopontin is an integrin-binding protein, implicated in cell-matrix interactions. It is overexpressed in several types of cancers (50), including MPM (29). Pass et al. analyzed serum samples from 69 asbestos-exposed subjects, 45 smoking subjects and 75 MPM patients. The duration of asbestos exposure independently correlated with Osteopontin serum levels (Figure 1). Furthermore, serum Osteopontin was higher in MPM patients than in asbestos-exposed controls (133 ± 10 vs. 30 ± 3 ng/ml). Receiver Operating Characteristic (ROC) analysis demonstrated that the most accurate cut-off value for stage I disease was 62.4 ng/ml, with 84.6% sensitivity and 88.4% specificity. Unfortunately, in another study serum Osteopontin failed to distinguish MPM patients from patients with pleural metastasis of different carcinomas or from subjects with non-tumoral asbestos-related diseases (25). Another study suggested that plasma Osteopontin is a more stable and reliable marker than serum Osteopontin; nevertheless, conclusive data about its diagnostic accuracy are still lacking (30, 31, 51). Combined assessment of SMRP and Osteopontin, was not more informative than SMRP alone (30, 31, 45, 52) (Table 1).

### Fibulin-3

Research for circulating biomarkers of MPM also included Fibulin-3 as a single biomarker or in combination with Mesothelin. Fibulin-3 is a secreted glycoprotein implicated in cell proliferation and migration (34). In the first report, plasma levels of Fibulin-3 were measured in a total of 92 MPM patients, 136 healthy asbestos-exposed controls and 93 patients affected by other cancers. The subjects enrolled in the study belonged to two different cohorts. Fibulin-3 was found to be higher in MPM patients compared to control groups; this alteration was not related to duration of asbestos exposure, age, sex, histologic subtype or tumor stage (32). ROC analysis showed an AUC of 0.99 and the best cut-off value was 52.8 ng/ml for all MPM patients and 46 ng/ml for stage I/II disease. Based on these results the authors concluded that Fibulin-3 was the best biomarker analyzed so far. However, in an analysis of a validation cohort comprising 48 MPM patients and 96 asbestos-exposed controls, the accuracy of Fibulin-3 did not differ from that reported for Mesothelin (AUC 0.87) (32). This discrepancy between training and validation sets may be due to differences in the cohorts analyzed (36, 53). Other studies indicated that Fibulin-3 was not useful for discriminating MPM patients from patients affected by other diseases (33), and did not perform as well as Mesothelin (54) (Table 1).

### Inflammatory and Angiogenic Factors

Chronic inflammation is considered a key determinant of MPM carcinogenesis (55). Inhaled asbestos fibers accumulate in the pleura and activate an inflammatory response. As macrophages cannot eliminate these fibers, inflammatory cytokines and growth factors are continuously produced, promoting malignant transformation. Tumor-associated macrophages (TAM) also produce, and induce production of cytokines and growth factors that enhance tumor growth and invasiveness (56).

The tight link between inflammation and cancer aggressiveness is supported by several studies demonstrating that the neutrophil-to-lymphocyte ratio (NLR), an indicator of systemic inflammation, is an independent predictor of poor prognosis in several cancers, including MPM (57–59). Based on this knowledge, the evaluation of inflammation markers was proposed for the diagnostic/prognostic stratification of MPM patients.

High Mobility Group B 1 (HMGB1) belongs to the family of damage-associated molecular pattern proteins (DAMPs) and is considered a key mediator of asbestos-induced inflammation (60, 61). In physiological conditions, HMGB1 is localized in the nucleus, where it functions as a chromatin-binding protein and is released by cells undergoing necrosis. In pathological conditions, myeloid cells and cancer cells can actively secrete a hyper-acetylated form of HMGB1. In the extracellular space, HMGB1 activates innate and adaptive immunity and acts as a pro-oncogenic factor binding to Toll like Receptors (TLRs) and RAGE (receptor of advanced glycation end products) (62). Jube et al. demonstrated that HMGB1 and its receptors are highly expressed in MPM tissues and cell lines. Exposure of normal mesothelial cells to asbestos induces necrosis, resulting in release of HMGB1 (Figure 1). Transformed MPM cells actively secrete acetylated HMGB1, which promotes cell proliferation and invasiveness in an autocrine manner (61). Consistent with this notion, Napolitano et al. showed that asbestos-exposed subjects (*N* = 42) had higher serum levels of total HMGB1 compared to non-exposed control (*N* = 20). In healthy exposed individuals (*N* = 20) the majority of serum HMGB1 was in the non-acetylated form (90%), while in MPM patients (*N* = 22) the acetylated form was prevalent (67%). ROC analysis showed that serum levels of acetylated HMGB1 discriminated healthy exposed controls from MPM patients with high accuracy (cut-off = 2 ng/ml; AUC = 1; 100% specificity, 100% sensitivity). Importantly, tumor stage did not influence acetylated HMGB1 levels (35) (Table 1). Although these results were obtained with a small number of subjects, they provide groundwork for future investigations on larger cohorts aimed at validating acetylated HMGB1 as an early diagnostic marker.

The angiogenic factor VEGF, a key stimulator of tumor neoangiogenesis, is overexpressed in MPM tissues (63–65). VEGF levels are also increased in pleural effusions (PE) of MPM patients compared to patients affected by non-malignant pleural diseases or lung cancer (66). Yasumitsu et al. showed that serum VEGF was higher in MPM patients (*N* = 51) compared to control patients with non-tumoral asbestos-related diseases (*N* = 29). Setting a cut-off at 460 pg/ml, these authors showed a strong correlation between high serum VEGF and shorter patient survival (67). A predictive/prognostic role of VEGF in MPM has also been described. Baseline serum levels of VEGF-A and VEGF receptor 2 (VEGFR-2) correlated with radiological response in patients treated with the multitarget tyrosine kinase inhibitor Sunitinib Malate (68). In patients with high baseline serum levels of VEGF, its decrease after 8 weeks of thalidomide treatment correlated with longer patient survival (69). Although these results are promising, they should be considered cautiously. Serum VEGF may not really reflect its circulating levels because it may be released by platelets during *in vitro* blood clotting (70). Considering that platelet count is an independent prognostic factor for MPM patient survival (71), VEGF should probably be evaluated in plasma instead of serum samples.

### Markers of Oxidative Stress

Reactive oxygen species (ROS) and reactive nitrogen species (RNS) are key mediators of asbestos toxicity (72). ROS and RNS are generated by asbestos through two main mechanisms. First, the different forms of asbestos fibers contain iron, which increases the generation of hydroxyl radicals through the reactions of Fenton and Haber-Weiss (73, 74). Consistent with this notion, X-ray imaging and spectroscopy studies showed that asbestos fibers in tissues contain iron in the form of ferritin and haematite (75). Second, inhaled asbestos fibers are internalized by alveolar epithelial cells (AEC) and alveolar macrophages (AM); the activation and attempted phagocytosis by AM and neutrophils lead to activation of vacuolar NADPH oxidase and myeloperoxidase, which generate ROS and hypochlorite radicals in the microenvironment. Undigested asbestos fibers are coated with a mucopolysaccharide, generating the pathognomonic asbestos bodies, and with iron protein complexes, resulting in the ferruginous bodies, which further enhance ROS production and local inflammation.

Bronchoalveolar lavage fluid (BALF) of asbestos-exposed patients (*N* = 14) exhibited an increase in several markers of inflammation and altered iron and ROS homeostasis (i.e., iron, transferrin, transferrin receptors, lactoferrin, and ferritin) compared to unexposed controls (*N* = 10) and asbestos-exposed subjects (*N* = 14) (76) (Figure 1). It will be interesting to test ROS-related markers in the peripheral blood of MPM patients and asbestos-exposed individuals.

## Circulating microRNAs (miRNAs)

miRNAs are small non-coding RNAs that regulate the expression of a vast number of mRNAs (77). Tumor cells exhibit distinctive miRNA signatures (78, 79) and can release miRNAs as a result of cell death and active secretion (80). Such cell-free circulating miRNAs (cfmiRNA) are relatively stable, since they are incorporated into membrane-bound vesicles or bound to ribonucleoprotein complexes (81).

Studies of cfmiRNAs are curbed by major problems in data normalization, given the difficulties in identifying “bona fide” housekeeping miRNA in biological fluids that can be used for normalization. Biases linked to the choice of an appropriate reference can be circumvented by using the miRNA ratio approach (82, 83), which is based on the calculation of ratios between upregulated and downregulated miRNAs in the same patient.

Several studies investigated the cfmiRNA profile in mesothelioma patients with the aim of identifying markers for early diagnosis and prognostic stratification (84). This section focuses mainly on miR-126-3p, miR-103a-3p, and miR-625, 3 miRNAs that appear to be consistently altered in MPM patients (Table 2).

**Table 2:** Circulating miRNAs in MPM.

**miRNAs**	**miRNA expression in MPM**	**Study design**	**Samples**	**Reference gene, method of analysis**	**Study results**	**References**
miR-126-3p	Reduced	MPM = 10, NMT = 5 (frozen biopsy); MPM = 27, adjNCT = 27 (FFPE); MPM = 44, HC = 50, AEXP = 196;	Frozen Biopsy, FFPE, tissue, Serum	Ref gene: RNU6 Method: TaqMan MicroRNA Assay	MPM vs. AEXP sensitivity: 73%, specificity: 74% AEXP vs. HC sensitivity: 60%, specificity: 74%	(85)
miR-126-3p	Reduced	MPM = 45, HC = 56, NSCLC = 20;	Serum	Ref gene: RNU6, cel-miR-39 Method: TaqMan microRNA Assay	MPM vs. HC sensitivity: 80%, specificity: 60%	(86)
miR-126-3p	Reduced	MPM = 45, AEXP = 99, HC = 44 (discovery group); MPM = 18, AEXP = 50, HC = 20, LC = 42 (validation group);	Serum	Ref gene: RNU6, cel-miR-39, Method: TaqMan MicroRNA Assay Circulating methylated TM DNA assay and ELISA	MPM vs. HC miR-126-3p sensitivity: 75%, low specificity: 54% miR-126 + SMRP + Met-TM: AUC = 0.857	(87)
miR-16 miR-17 miR-126 miR-486	Reduced	MPM = 32, AEXP = 14, NCP = 15; 24 MPM (FFPE)	Plasma, FFPE Tissue	Ref gene: miRNA-146 for plasma RNU6B, RNU44, RNU48 for tissue Method: TaqMan microRNA Assay	MPM ± AEXP vs. NCP miR-16: AUC = 0.89, cut-off 77.5, sensitivity: 86.7%, specificity: 82.2% miR-17: AUC = 0.88, cut-off 5.9, sensitivity: 80%, specificity: 84.4% miR-126: AUC = 0.95, cut-off 5.4, sensitivity: 80%, specificity: 97.8% miR-486 AUC = 0.88, cut-off 9.2, sensitivity: 80%, specificity: 89.1%	(88)
miR-126-3p miR-132-3p miR-103a-3p	Reduced	MPM = 17, AEXP = 34;	Plasma, Blood Cells	Ref gene: RNU6 for miR-126-3p, miR-146b-5p for miR-132-3p, miR-125a for miR-103a-3p Method: TaqMan microRNA Assay; miR-103a in whole blood cell fraction	MPM vs. AEXP miR-126-3p: AUC = 0.614, sensitivity: 0%, specificity: 98% miR-132-3p: AUC = 0.542, sensitivity: 0%, specificity: 98% miR-103a-3p: AUC = 0.603, sensitivity: 0%, specificity: 98% miR-126-3p + miR-132-3p + miR-103a-3p: AUC = 0.605, sensitivity: 0%, specificity: 98%	(89)
miR-625-3p	Increased	MPM = 5, HC = 3 (plasma); MPM = 15, CS = 14 (plasma); (test cohort) MPM = 30, AEXP = 10 (serum); (validation cohort) MPM = 18, CS = 7 (FFPE);	Plasma, Serum, FFPE Tissue	Ref gene: miR-16 (plasma), RNU6B (FFPE) Method: Human miRNA, Microarray Agilent; TaqMan miRNA Assay, OpenArray Analysis	MPM vs. AEXP test cohort AUC = 0.824 sensitivity: 73.33% specificity: 78.57% MPM vs. AEXP validation cohort AUC = 0.793 sensitivity: 70% specificity: 90%	(90)
miR-103a-3p	Reduced	MPM = 23, AEXP = 17, HC = 25;	Blood cells	Ref gene: miR-125a Method: miRNA Microarray, TaqMan miRNA Assay	MPM vs. AEXP AUC = 0.757, cut-off 0.621 sensitivity: 83% specificity: 71% MPM vs. HC AUC = 0.871, cut-off 0.621 sensitivity: 78% specificity: 76%	(91)
miR-103a-3p	Reduced	MPM = 43, AEXP = 52;	Blood cells	Ref gene: miR-125a. Method: TaqMan miRNA Assay	MPM vs. AEXP miR-103a-3p: AUC = 0.76, cut-off 749.61 sensitivity: 86% specificity: 63% miR-103a-3p + Mesothelin: AUC = 0.90, sensitivity: 86% specificity: 85%	(92)
miR-103a-3p, miR-30e-3p	Reduced	MPM = 23, AEXP = 19;	Extra-Cellular Vescicles (EV)	Ref gene: RNU48, average of miR-99a, miR-638, miR-720, miR-1274a. Method: OpenArray qRT-PCR, Custom TaqMan™ Low Density Array	MPM vs. AEXP miR-103a-3p + miR-30e-3p: AUC = 0.942 sensitivity: 95.5% specificity: 80%	(93)
miR-2053	Increased	MPM = 100, AEXP = 20, HC = 20;	Serum	Ref gene: RNU6B (serum), ACTB (RNAs) Method: miScript SYBR Green PCR, miScript Primer Assay	MPM vs. HC miR-2053: AUC = 0.91, cut-off 1.25 sensitivity: 85 % specificity: 97.5 % miR-2053 + lnc-RP1-86D1.3 + ARSA + DRAM1: AUC = 0.94, sensitivity: 100 %, specificity: 85%	(94)

*MPM, malignant pleural mesothelioma; NSCLC, non-small cell lung cancer; LC, lung cancer; CS, control subjects (patients with coronary artery disease or healthy subjects); AEXP, asbestos exposed patients; HC, healthy volunteers; NCP, controls with noncancerous pulmonary diseases; SMRP, soluble mesothelin-related proteins; Met, TM-methylated thrombomodulin promoter; adjNCT, adjacent non-cancerous tissue; NMT, non-malignant tissue*.

Santarelli et al. showed that miR-126-3p is strongly downregulated in serum samples from MPM patients (*N* = 44) compared to samples from healthy volunteers (*N* = 50) or asbestos-exposed subjects (*N* = 196) (85). ROC curve analysis indicated that this miRNA distinguished MPM patients from asbestos-exposed individuals with 73% sensitivity and 74% specificity (85). The combined upregulation of soluble SMRP and downregulation of miR-126-3p was associated with a high risk of mesothelioma development. However, these data were normalized using the small nucleolar RNA RNU6 (U6), which is known to be present at low and variable levels in blood (83) and may be altered in chronic inflammation (95), which is very common in asbestos-exposed individuals.

Tomasetti et al. (86) confirmed that miR-126-3p discriminates MPM patients (*N* = 45) from healthy controls (*N* = 56) (sensitivity 80%, specificity 60%) and that its levels are lower in MPM patients with poor prognosis compared to those with better clinical outcome and to patients with non-small cell lung cancer (*N* = 20). In this study, the samples were normalized to spiked-in cel-miR-39, endogenous U6 or both (86).

Interestingly, the diagnostic performance of miR-126-3p was significantly improved when combined with Mesothelin and methylation of the thrombomodulin promoter (AUC 0.857, 95% CI 0.767–0.927) (87) (see also section on DNA methylation).

In apparent contrast with these studies, Mozzoni et al. (88) did not confirm the ability of miR-126-3p to discriminate MPM patients (*N* = 32) from asbestos-exposed controls (*N* = 14), and did not observe a correlation between the levels of miR-126-3p in the plasma and in the MPM tissues (*N* = 24). However, miR-126-3p was able to distinguish MPM patients (*N* = 32) and asbestos-exposed patients (*N* = 14) from control subjects with non-cancerous pulmonary diseases (*N* = 15). It must be noted that in this study, the authors used different normalizer RNAs for plasma (miR-146) and tissue (U6, RNU44, RNU48). More recently, Weber et al. (89) analyzed the levels of miR-126-3p, miR-132-3p, and miR-103a-3p in plasma samples obtained a median of 8.9 months prior to the diagnosis of MPM (*N* = 17), and compared them to asbestos-exposed controls (*N* = 34). This study indicated 0% sensitivity of these miRNAs considering a specificity of 98%. Based on these findings, the authors concluded that these miRNAs are unsuitable as biomarkers for early detection of MPM in asbestos-exposed individuals. However, it must be noted that, to permit a comparison with previous studies, the authors normalized miR-126-3p against U6, miR-132-3p against miR-146b-5p, and miR-103a-3p against miR-125a (89). As the authors suggest, it would be desirable to employ a common normalizer for all these miRNAs.

Kirschner et al. demonstrated higher levels of miR-625-3p in the serum of MPM patients (*N* = 30) compared to asbestos-exposed subjects (*N* = 10) (accuracy 79.3%, sensitivity 70% and specificity 90%) (90). However, these data were normalized against miR-16, which is known to be highly dependent on the haemolysis of the sample (90) and was also reported to be altered in MPM (88, 96).

Weber et al. took a different approach and analyzed the cell fraction obtained by centrifugation of whole blood; in this fraction miR-103a-3p was downregulated in MPM patients (*N* = 23) compared to asbestos-exposed (*N* = 17) and healthy control subjects (*N* = 25). miR-103a-3p discriminated MPM patients from asbestos-exposed subjects with a 83% sensitivity and 71% specificity, and from healthy controls with 78% sensitivity and 76% specificity (91). In a subsequent study the authors confirmed this finding and provided evidence that the association of reduced levels of miR-103a-3p in blood cells with elevated Mesothelin in plasma improved the discrimination of MPM patients (*N* = 43) from asbestos-exposed (*N* = 52) individuals (92). However, these findings were not confirmed in a follow-up study of prediagnostic MPM samples (89). In all these studies data were normalized to miR-125a measured in the cell fraction of whole blood.

Cavalleri et al. analyzed the levels of miR-103a-3p along with miR-30e-3p in extracellular vesicles and showed that the combination of these two markers discriminated MPM patients from asbestos-exposed subjects with a 95.5% sensitivity and 80% specificity. These findings were confirmed by normalizing the data to RNU48, miR-99a, miR-638, miR-720, and miR-1274a (93).

A recent study by Matboli et al. (94) detected increased levels of the long non-coding-RNA RP1-86D1.3 and miR-2053 and downregulation of the mRNAs coding for DRAM1 (damage-regulated autophagy modulator) and ARSA (arylsulfatase A) in MPM patients (*N* = 100) compared to asbestos-exposed subjects (*N* = 20) and healthy controls (*N* = 20). Data were normalized to RNU6B for miR-2053 and to beta actin for the other RNAs. The function of long non-coding-RP1-86D1.3 is obscure at present, although its expression is frequently altered in lung, breast, colon, and gastric cancer (97, 98). DRAM1 is a p53 responsive gene that encodes a lysosomal membrane protein involved in autophagy (99). ARSA is a lysosomal enzyme that is necessary for the correct function of autophagosomes (100). These mRNAs could be considered as biomarkers of asbestos exposure rather than disease. Moreover, the authors suggest that the upregulation of miR-2053 is a good prognostic marker of MPM, which will be validated in a large sample cohort (94).

## Circulating Tumor DNA (ctDNA)

ctDNA comprises the fraction of circulating cell-free DNA (cfDNA) that is released by the tumor through apoptosis, necrosis, or active tumor secretion (13). cfDNA is typically found as double-stranded fragments measuring 180-200 base pairs in length, corresponding to nucleosome-associated DNA (101, 102). Cancer patients commonly exhibit a higher concentration of cfDNA (103) that contains the mutations found in the tumor.

The detection of ctDNA variants in MPM holds promise as a potential biomarker for the diagnosis and stratification of MPM patients.

Sriram et al. showed that the DNA integrity index (i.e., the ratio between ALU fragments of 247 and 115 bp) in pleural fluid was higher in MPM patients (*N* = 16) than in benign pleural effusions (*N* = 23) (median: 1.2 vs. 0.8 with *p* < 0.001) (104). ROC analysis of this cohort revealed that serum Mesothelin had the highest predictive value (AUC: 0.94) followed by pleural fluid Mesothelin (AUC: 0.89) and DNA integrity index in pleural effusion (AUC: 0.82). Using a cut-off of 1.06 for the DNA integrity index, a cut-off of 12.91 nM for pleural fluid Mesothelin and a cut-off of 1.34 nM for serum Mesothelin, the authors obtained a specificity of 90% and a sensitivity of 75% to distinguish malignant pleural mesothelioma from benign pleural effusion. Further studies are needed to test whether the DNA integrity index in pleural fluid may provide additional information about the progression of disease.

A comprehensive genomic analysis conducted by Bueno et al. on a large cohort of MPM tissue samples revealed mutations in the BAP1, NF2, TP53, SETd2, DDX3X, ULK2, RYR2, CFAP45, SETDB1, and DDX51 genes (105). Using an integrated analysis, these authors identified alterations in the Hippo, mTOR, histone methylation, RNA helicase and p53 signaling pathways.

However, to date none of these mutations have been systematically investigated using a liquid biopsy approach. In a recent study, Hylebos et al. (106) performed whole exome sequencing (WES) of tumor and germline DNAs of ten MPM patients and confirmed the mutation described by Bueno et al. Selected tumor-specific variants of ctDNA were detected in serum samples using ddPCR (Droplet Digital PCR), but the mutation in NF2 was clearly and reproducible detectable in only one patient (fraction of mutated DNA = 0.8%).

## DNA Methylation

DNA methylation is an epigenetic modification that usually occurs at regions of DNA rich in CpG dinucleotides, which are located mainly in 5' regulatory regions of genes. The DNA methylation pattern may be modified following environment exposure, therapy, aging and disease. Studies have demonstrated that promoter methylation, and alterations of gene expression are a common occurrence in mesothelioma and that the DNA methylation profile in tissue samples was able to distinguish normal pleura from mesothelioma (107). Detection of changes in the methylation profile of ctDNA might thus be a tool for early diagnosis and prognostic stratification of MPM patients (108).

Santarelli et al. (87) evaluated alterations in the methylation of the thrombomodulin (TM) gene in serum in association with serum levels of SMRP and miR-126 in MPM patients (*N* = 45), asbestos-exposed healthy subjects (*N* = 99), and healthy donors (*N* = 44). The model based on the combination of these 3 parameters improved the differential diagnosis of MPM, with an AUC of 0.857. A significant risk of disease (odds ratio >4) was found in the presence of high levels of SMRP in association with altered levels of either miR-126 or TM promoter methylation, and when both miR-126 and TM promoter methylation were altered even at low SMRP concentration. It will be interesting to test the validity of these findings in large prospective longitudinal cohorts (87, 109).

In a very recent study, Guarrera et al. (110) investigated peripheral blood DNA methylation as a biomarker of MPM in a large cohort of patients and controls. Results showed a distinct methylation signature in MPM patients compared to controls, with more than 800 differentially methylated (DM) CpG sites and significant enrichment for genes controlling innate immunity and neutrophil degranulation. The authors identified seven top DM-CpGs, three of which were hypomethylated (FOXK1, MYB, and TAF4) and four hypermethylated (CXCR6/FYCO1, TAP1, MORC2, and LIME1). ROC analysis showed a diagnostic value of the methylation levels of the seven top DM-CpGs in association with age, sex and asbestos exposure levels (AUC: 0.89). Univariate regression analysis showed no clear evidence for differences in the seven DM-CpGs among the different histotypes of mesothelioma. Overall, the results obtained in these studies are very promising but need to be validated in a longitudinal study.

## Circulating Tumor Cells (CTCs)

CTCs are intact tumor cells derived from primary or metastatic tumor sites. The number of CTCs present in the blood is very low at early stages and increases in advanced stages of cancer (111).

To date, CELLSEARCH® is the only FDA-approved test for capturing and counting CTCs. This method consists of magnetic particles coated with antibodies targeting the Epithelial Cell Adhesion Molecule (EpCAM), an antigen present on most epithelial tumor cells (112).

For MPM, which originates from the mesothelium, the CELLSEARCH technique has demonstrated a very low diagnostic sensitivity (113–115).

More recently, Chikaishi et al. developed a “CTC-chip” that was able to capture the Ep-Cam negative CTCs by targeting podoplanin (116), a mucine-type transmembrane glycoprotein whose expression is increased in malignant cells of mesothelial origin (117). Yoneda et al. (118) evaluated CTCs in a small cohort of 16 MPM patients using the CellSearch and CTC-chip techniques. The CTC-chip performed better than CellSearch, and demonstrated a significant diagnostic value in discriminating between early and advanced disease (AUC = 0.851).

## Conclusions and Perspectives

At present, there is no reliable marker for the longitudinal monitoring and risk assessment of asbestos-exposed individuals. Although liquid biopsy is still far to replace tissue biopsy for MPM diagnosis, it holds great promise for non-invasive tracking of the follow-up of asbestos-exposed individuals. Plasma and serum samples represent minimally invasive, low risk, and easily obtained biological fluids and many studies have indicated potentially interesting biomarkers, including Mesothelin (early diagnosis and prognostic stratification of MPM), Osteopontin (early diagnosis), Fibulin-3 (early diagnosis), HMGB1 (early diagnosis), VEGF (early diagnosis and prognostic stratification) and miRNAs (early diagnosis). More recent studies have also suggested that markers of oxidative stress, CTCs and ctDNA might also be useful for the screening/early diagnosis of MPM. Furthermore, a study by Zucali et al. demonstrated that TS (Thymidylate Synthase) is overexpressed in MPM tissues and is a strong predictor of responsiveness of MPM patients to Pemetrexed/Carboplatin (119). It is thus possible that detection of TS in circulating MPM cells or as circulating cell-free RNA might prove to be an interesting predictive biomarker (120, 121).

However, most of these markers were studied in restricted patients' cohorts, and the conclusive identification of robust circulating biomarkers for early diagnosis and prognostic stratification of MPM patients awaits validation in large prospective studies. Furthermore, most of these studies were highly heterogeneous in terms of preanalytical and analytical protocols employed. Therefore, future efforts should be focused on reaching a consensus on the standardization and normalization of the different assays to achieve robust and reproducible results. Multivariate analyses of multiple biomarkers may also improve the diagnostic power of these assays.

## Author Contributions

IC prepared the paragraphs on ctDNA, DNA methylation, CTC, and revised the final version of the paper. LU prepared the paragraph on Circulating Tumor Proteins and revised the final version of the paper. ES prepared the paragraph on Circulating miRNAs and revised the final version of the paper. GP prepared the Introduction and revised the final version of the paper. VC prepared the paragraphs on Oxidative Stress Markers, on Perspectives, and revised the final version of the paper.

### Conflict of Interest Statement

The authors declare that the research was conducted in the absence of any commercial or financial relationships that could be construed as a potential conflict of interest.
